# Rapid Increase in Serum Low-Density Lipoprotein Cholesterol Concentration during Hepatitis C Interferon-Free Treatment

**DOI:** 10.1371/journal.pone.0163644

**Published:** 2016-09-28

**Authors:** Satoru Hashimoto, Hiroshi Yatsuhashi, Seigo Abiru, Kazumi Yamasaki, Atsumasa Komori, Shinya Nagaoka, Akira Saeki, Shinjiro Uchida, Shigemune Bekki, Yuki Kugiyama, Kazuyoshi Nagata, Minoru Nakamura, Kiyoshi Migita, Kazuhiko Nakao

**Affiliations:** 1 Clinical Research Center, National Hospital Organization (NHO) Nagasaki Medical Center, Nagasaki University Graduate School of Biomedical Sciences, Nagasaki, Japan; 2 Department of Gastroenterology and Hepatology, Graduate School of Biomedical Sciences, Nagasaki University, Nagasaki, Japan; Chiba University, Graduate School of Medicine, JAPAN

## Abstract

**Background & Aim:**

We performed lipid analyses at the early period of therapy in patients with chronic hepatitis C who underwent interferon (IFN)-free direct-acting antiviral (DAA) treatment, and we attempted to identify the factors that contributed to a rapid increase in the patients’ serum low-density lipoprotein cholesterol (LDL-C) concentration.

**Methods:**

We retrospectively analyzed the cases of 100 consecutive patients with HCV infection treated at the National Hospital Organization Nagasaki Medical Center: 24 patients underwent daclatasvir (DCV) and asunaprevir (ASV) combination therapy (DCV/ASV) for 24 weeks, and the other 76 patients underwent ledipasvir and sofosbuvir combination therapy (LDV/SOF) for 12 weeks. ΔLDL-C was defined as the changed in LDL-C level at 28 days from the start of therapy. To determine whether ΔLDL-C was associated with several kinds of factors including viral kinetics, we performed a stepwise multiple linear regression analysis.

**Results:**

The LDL-C levels in patients treated with LDV/SOF were markedly and significantly elevated (87.45 to 122.5 mg/dl; p<10^−10^) compared to those in the DCV/ASV-treated patients (80.15 to 87.8 mg/dl; p = 0.0056). The median levels of ΔLDL-C in the LDV/SOF and DCV/ASV groups were 33.2 and 13.1, respectively. LDV/SOF combination therapy as an IFN-free regimen (p<0.001) and ΔHCV core antigen (0–1 day drop) (p<0.044) were identified as independent factors that were closely related to the ΔLDL-C.

**Conclusions:**

A rapid increase in the serum LDL-C concentration during the IFN-free treatment of hepatitis C was associated with the type of HCV therapy and a decline of HCV core protein.

## Introduction

Hepatitis C virus (HCV) is a major causative agent of chronic liver diseases including steatosis, cirrhosis, and hepatocellular carcinoma. It has been estimated that worldwide there are 170 million patients with chronic liver disease, of whom most are infected with HCV [1–3]. The patients with chronic HCV infection can be associated with fatty liver, hypobetalipoproteinemia, and hypercholesterolemia as a reason for the lipid and lipid protein metabolism disorders [4]. Some studies indicated patients with chronic HCV infection had the high risk of ischemic heart diseases such as angina pectoris and myocardial infarction [5,6]. The patients with chronic HCV genotype-3 infection had a greater chance of a fatty liver than other genotypes and decreased serum low density lipoprotein (LDL) [4,7]. HCV genotype 1b has been the dominant genotype in Japan [8].

Recent research into the HCV life-cycle has demonstrated a strong interaction between the virus and intracellular lipids, suggesting that host lipids play an important role in viral replication. Host serum lipids play a role in hepatitis C virion circulation and hepatocyte entry. A proportion of circulating hepatitis C viral particles are complexed with host triacylglycerol-rich lipoproteins, known as lipo-viroparticles [9–11]. Lipo-viroparticles use LDL receptors on hepatocytes as points of entry and are associated with high rates of infectivity [11–13]. Mazumdar *et al*. reported that the interaction of envelope glycoprotein 1 with apolipoprotein may promote an entry into hepatocytes [14]. Once hepatitis C virions have entered hepatocytes, their replication is again dependent on host lipid interactions. New hepatitis C virion formation requires viral binding to either an endoplasmic reticulum phospholipid membrane or to an endoplasmic reticulum-associated membranous web [15].

In Japan, the treatment of chronic HCV infection is now possible with interferon (IFN)-free regimens composed of direct-acting antiviral (DAA) agents that directly inhibit viral proteins [16–19]. Marked advances in the IFN-free DAA treatment of HCV infection in patients with chronic hepatitis C have also been reported, indicating that HCV disease will be increasingly eradicated in this population [16–19].

IFN-free DAA treatment for HCV in elderly Japanese HCV-infected patients was reported to achieve a sustained virologic response (SVR), but it was not known how these patients’ lipid profiles were altered after the SVR was achieved [16,19]. We found two studies that described a marked increase in serum low-density lipoprotein cholesterol (LDL-C) in HCV mono-infected or HCV/HIV co-infected patients treated with sofosbuvir and ribavirin (SOF/RBV) or ledipasvir/sofosbuvir combination therapy that correlated with a viral decline in serum, suggesting a direct influence of HCV clearance on serum cholesterol [20,21].

In the present study, we performed lipid analyses in patients with chronic hepatitis C who underwent IFN-free DAA treatment at the early period of therapy, and we attempted to identify the factors that contribute to a rapid increase in the serum LDL-C concentration.

## Patients and Methods

### Patients

Between January 2013 and December 2015, a total of 100 consecutive HCV genotype 1 patients who underwent an IFN-free regimen at the National Hospital Organization Nagasaki Medical Center were enrolled in our retrospective study. The diagnosis of chronic infection of HCV was based on the finding of continuous positivity for anti-HCV and HCV-RNA by polymerase chain reaction (PCR). The clinical condition of each patient’s liver was defined using the FIB-4 index. The presence of advanced liver disease was diagnosed by cirrhotic change in the liver shape by ultrasonography, computed tomography or magnetic resonance imaging and an FIB-4 index >3.25, which predicts the existence of significant fibrosis (F3–F4) [22]. Patients who were regularly prescribed lipid-lowering agents were not enrolled in this study.

### Treatment Regimen

Antiviral therapy without IFN against HCV was administered to all patients. Among the 100 patients, 24 received the combination therapy of daclatasvir (DCV, 60 mg once daily) and asunaprevir (ASV, 100 mg, twice daily) for 24 weeks (DCV/ASV). The other 76 patients received the combination therapy of ledipasvir (LDV, 90 mg, once daily) and sofosbuvir (SOF, 400 mg daily) for 12 weeks (LDV/SOF). LDV/SOF was available as a fixed-dose combination tablet named Harvoni® (Gilead Sciences, Foster City, CA).

### Laboratory Testing

For all patients in our cohort, a blood sample was taken at three time points: the day of IFN-free treatment start (D0), 24 hr after the first administration of an antiviral agent (D1), and 4 weeks after the start of therapy (D28). We retrieved each patient’s medical history along with the results of routine tests for blood cell counts, liver biochemistry and HCV viral load at the time of treatment and thereafter, from their medical records. Complete blood counts and biochemical tests including total cholesterol (TC), triglyceride (TG), and LDL-C were performed at D0, D1, and D28 using automated procedures in the clinical laboratories of our hospital. ΔTC and ΔLDL-C were defined as the change in each parameter from D0 to D28.

### HCV Quantification

We measured the HCV viral load by performing two assays simultaneously: HCV-RNA and HCV core antigen assays. We determined the HCV-RNA by conducting a TaqMan PCR using a commercial kit (COBAS® TaqMan® HCV Auto, Roche Diagnostics, Mannheim, Germany), with the measurement range 1.2–7.8 (log_10_IU/ml). HCV core antigen was determined by the chemiluminescent immunoassay (CLIA) method (ARCHITECT ® HCV Ag, Abbot Japan, Tokyo), with the measurement range 3.0–180,000 (fmol/L). We measured the viral load by determining both the HCV-RNA and HCV core antigen values at D0, D1, and D28. For 17 of the patients, there was loss of HCV core antigen data at D1, and were therefore analyzed the viral decline by both assays in only 83 patients. The definition of ΔHCV-RNA and ΔHCV core antigen were the change of each observed value from D0 to D1.

### Ethical Consideration

Written informed consent for the use of their medical records, stored sera and liver specimens was obtained from each patient. This study was conducted according to the ethical guidelines of the Declaration of Helsinki, and was approved by the Ethics Committee of the National Hospital Organization Nagasaki Medical Center (confirmation no.: 27155).

### Statistical Analysis

Categorical data (gender, clinical condition of liver) were compared by chi-square test. The continuous variables (age, body mass index [BMI], HCV-RNA, HCV core antigen, aspartate aminotransferase [AST], alanine aminotransferase [ALT], gamma-glutamyl transpeptidase [γ-GTP], albumin, platelet count, TC, LDL-C, TG and alpha fetoprotein [AFP]) were dichotomized with respect to the median value or clinically meaningful values in a multivariate analysis. P-values <0.05 were considered significant. All statistical analyses were performed using the Statistical Package for the Social Sciences software program, ver. 23.0 (SPSS, Chicago, IL).

## Results

### Patient Characteristics

The characteristics of the patients at baseline are summarized in Table 1. Of the 100 patients, 24 received DCV/ASV, and the remaining 76 received LDV/SOF. The median age of all patients was 71.0 years. The gender distribution was 39 (39%) males and 61 (61%) females. Seventy (70%) patients had advanced liver disease defined as an FIB-4 index >3.25. The median level of HCV-RNA was 6.0 (interquartile range [IQR]: 5.6, 6.4 log_10_IU/ml) and that of HCV core antigen was 3.6 (IQR 3.3, 4.0 log_10_ fmol/L). The median pretreatment value of TC was 155.5 (IQR 131.5, 185.0 mg/dl); that of LDL-C was 84.5 (IQR 68.4, 103.9 mg/dl), and that of TG was 96.0 (IQR 69.5, 128.3 mg/dl).

**Table 1:** Baseline Demographics and Clinical Characteristics of Patients.

Characteristics	Total	DCV/ASV	LDV/SOF	P-value†
	(n = 100)	(n = 24)	(n = 76)	
Age, year	71.0 (63.5, 76.0)	76.0 (69.0, 80.0)	69.0 (63.0, 75.0)	<0.01
Sex, Male, n(%)	39 (39.0)	8 (33.3)	31 (40.8)	0.51
BMI (kg/m^2^)	22.9 (21.0, 25.0)	23.1 (20.9, 25.9)	22.9 (21.0, 24.8)	0.54
Fib-4 index, 3.25≦, n (%)	70 (70.0)	19 (79.2)	51 (67.1)	0.26
HCV-RNA (log_10_IU/ml)	6.0 (5.6, 6.4)	6.1 (5.4, 6.4)	6.0 (5.6, 6.4)	0.64
HCV core antigen (log_10_(fmol/L))	3.6 (3.3, 4.0)	3.5 (3.2, 3.9)	3.6 (3.3, 4.0)	0.40
AST (IU/L)	49.0 (35.0, 64.5)	49.0 (34.5, 67.5)	49.0 (35.5, 63.0)	0.91
ALT (IU/L)	38.0 (26.0, 55.0)	36.0 (18.0, 57.0)	38.0 (28.0, 53.5)	0.15
γ-GTP (IU/L)	29.5 (20.0, 42.5)	29.5 (19.5, 44.0)	29.5 (20.5, 42.0)	0.97
Alb (g/dl)	3.9 (3.5, 4.3)	3.9 (3.5, 4.2)	3.9 (3.5, 4.4)	0.70
Platelet count (×10^3^/μl)	125 (90, 153)	122 (94, 144)	126 (90, 157)	0.86
TC (mg/dl)	155.5 (131.5, 185.0)	149.0 (133.0, 168.0)	157.5 (131.0, 186.0)	0.48
LDL-C (mg/dl)	84.5 (68.4, 103.9)	80.2 (65.3, 94.6)	87.5 (68.6, 105.8)	0.28
TG (mg/dl)	96.0 (69.5, 128.3)	86.0 (61.5, 110.5)	98.0 (71.3, 143.0)	0.08
AFP (ng/ml)	6.0 (4.0, 11.5)	6.1 (5.4, 6.4)	5.0 (4.0, 11.0)	0.07

Continuous variables are shown as median (with IQR) with analysis by Mann-Whitney’s test.

Categorical variables are expressed as number of patients (n) with frequencies (%), with analysis by Chi-squared test.

†DCV/ASV v.s LDV/SOF

### Changes in Lipid-Related Markers

**Fig 1:**
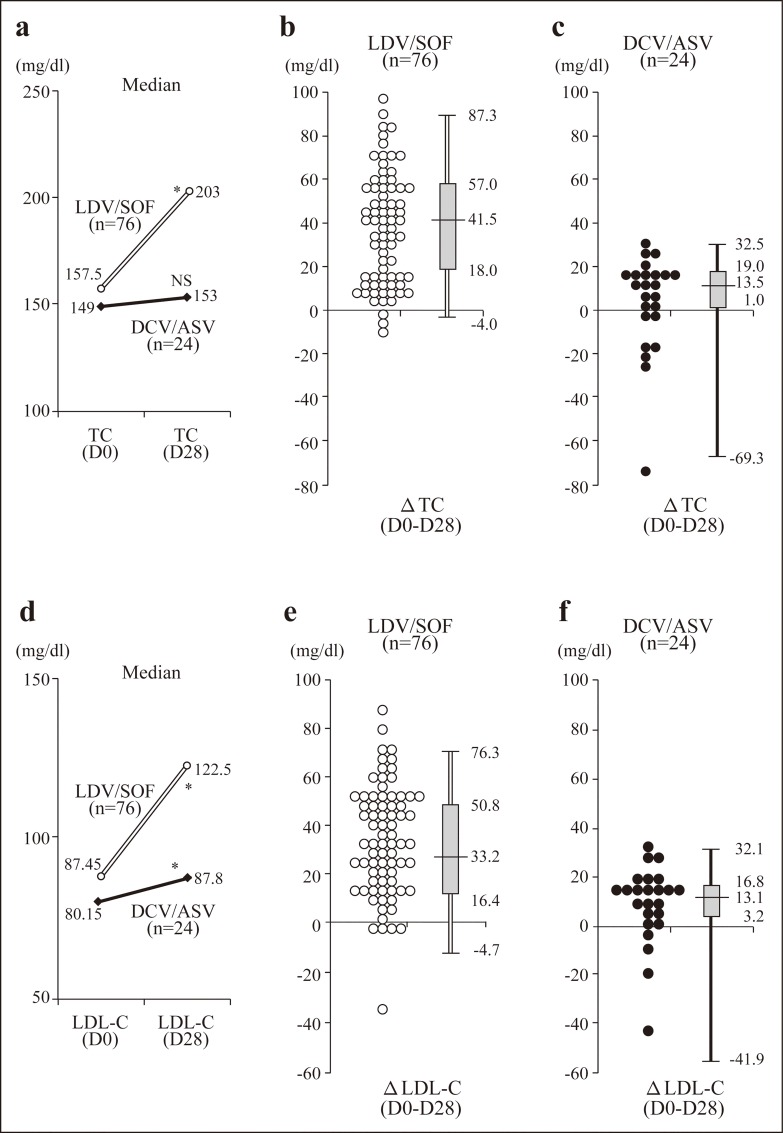
Scatterplots and boxplots of ΔTC or ΔLDL-C levels after the administration of the IFN-free regimen. (a) Median changes of TC in each therapy. (b,c) Scatterplots and boxplots of ΔTC in the LDV/SOV patient group (b) and in the DCV/ASV group (c). (d) Median changes of LDL-C in each therapy. (e,f) Scatterplots and boxplots of ΔLDL-C in the LDV/SOF (e) and DCV/ASV (f) −groups. *p<0.001.

The dynamic changes in the patients’ TC and LDL-C levels during their IFN-free therapy are illustrated in Fig 1. The median TC value of the patients who received LDV/SOF was significantly increased by week 4 of the therapy (157.5 to 203.0 mg/dl; p<10^−9^) compared to that of the patients who received DCV/ASV (149.0 to 153.0 mg/dl; p = 0.054) (Fig 1A).

The median levels of ΔTC in the LDV/SOF and DCV/ASV patient groups were 41.5 (IQR 18.0, 57.0 mg/dl) and 13.5 (IQR 1.0, 19.0 mg/dl), respectively (Fig 1B and 1C). Similarly, the LDL-C levels in the patients treated with LDV/SOF were markedly and significantly elevated (87.45 to 122.5 mg/dl; p<10^−10^) compared to those in the DCV/ASV-treated patients (80.15 to 87.8 mg/dl; p = 0.0056) (Fig 1D). The median levels of ΔLDL-C in the LDV/SOF and DCV/ASV groups were 33.2 (IQR 16.4, 50.8 mg/dl) and 13.1 (IQR 3.2, 16.8 mg/dl), respectively (Fig 1E and 1F).

Patients were divided into two groups stratified by their ΔLDL-C levels. The high ΔLDL-C group was defined as having a ΔLDL-C level ≥25.0 mg/dl, using the median value of all subjects. The low ΔLDL-C group was thus defined as having a ΔLDL-C level <25.0 mg/dl. We performed a multiple logistic regression analysis to identify the factors associated with LDL-C elevation at 4 weeks from the start of therapy (Table 2). Our univariate analysis had identified ‘IFN-free regimen’ (i.e., the LDV/SOF regimen) and ‘ΔHCV core antigen’ as the factors associated with ΔLDL-C. According to the multivariate analysis, ‘IFN-free regimen’ (LDV/SOF) was identified as an independent factor that was closely related to the LDL-C elevation.

**Table 2:** Factors Associated with ΔLDL-C value with HCV administered IFN-free regimen, Analyzed by Multiple Logistic-regression Analysis.

Characteristics	High ΔLDL-C group	Low ΔLDL-C group	Univariate	Multivariate	OR (95% CI)†
	ΔLDL-C > = 25	ΔLDL-C <25	P-value	P-value	
	(n = 50)	(n = 50)			
Age, year	68.0 (64.0, 75.0)	72.5 (63.0, 79.0)	0.12	0.60	1.02 (0.94–1.11)
Sex, Male, n(%)	20 (40)	19 (38)	0.84	0.31	0.47 (0.11–2.01)
BMI (kg/m^2^)	22.9 (21.2, 24.3)	23.2 (20.5, 25.9)	0.60	0.77	0.97 (0.80–1.18)
Advanced liver disease, n (%)	31 (62)	39 (78)	0.08	0.42	3.00 (0.20–44.69)
IFN-free regimen, LDV/SOF, n (%)	47 (94.0)	29 (68.0)	<0.01	0.03	12.15 (1.33–110.94)
HCV-RNA (D0) (log_10_IU/ml)	6.0 (5.6, 6.3)	6.0 (5.6, 6.4)	0.80	0.22	5.20 (0.36–74.21)
HCV core antigen (D0) (log_10_(fmol/L))	3.6 (3.3, 3.9)	3.7 (3.3, 4.0)	0.37	0.21	0.15(0.01–2.92)
AST (IU/L)	48.5 (36.0, 63.0)	49.5 (35.0, 64.0)	0.89	0.18	0.94 (0.86–1.03)
ALT (IU/L)	40.0 (26.0, 61.0)	34.5 (25.0, 50.0)	0.13	0.15	1.05 (0.98–1.11)
γ-GTP (IU/L)	28.0 (20.0, 47.0)	30.5 (20.0, 41.0)	0.64	0.76	1.00 (0.98–1.03)
Alb (g/dl)	3.9 (3.6, 4.2)	3.9 (3.5, 4.3)	0.56	0.75	0.72 (0.10–5.15)
Platelet count (×10^3^/μl)	131 (98, 164)	117 (86, 139)	0.09	0.06	1.24 (0.99–1.55)
TC (mg/dl)	159.0 (131.0, 185.0)	152.5 (132.0, 179.0)	0.44	0.22	1.02 (0.99–1.06)
LDL-C (mg/dl)	86.9 (65.0, 103.3)	82.0 (70.3, 104.4)	0.93	0.14	0.97 (0.92–1.01)
TG (mg/dl)	104.0 (71.0, 144.3)	87.5 (69.0, 115.0)	0.09	0.92	1.00 (0.99–1.01)
AFP (ng/ml)	6.0 (4.0, 15.0)	5.5 (4.0, 10.0)	0.35	0.90	1.00 (0.97–1.03)
ΔHCV-RNA (D0-D1) (log_10_IU/ml)	2.6 (2.3, 2.9)	2.8 (2.4, 2.9)	0.38	0.13	0.25 (0.04–1.49)
ΔHCV core antigen (D0-D1) (log_10_(fmol/L))	1.8 (1.6, 2.1)	1.6 (1.3, 1.9)	0.02	0.05	5.56 (0.98–31.69)

Continuous variables are shown as median (with IQR) with analysis by Mann-Whitney’s test.

Categorical variables are expressed as number of patients (n) with frequencies (%), with analysis by Chi-squared test.

†OR: Odds ratio, CI: Confidence interval

We performed a step-wise multiple linear regression analysis to determine whether ΔLDL-C was associated with age, gender, IFN-free regimen, clinical condition of liver disease, HCV-RNA, HCV core antigen level, BMI, AST, ALT, γ-GTP, platelet count, AFP, TC, LDL-C, TG, ΔHCV core antigen or ΔHCV-RNA. The results are shown in Table 3. ‘IFN-free regimen’ (LDV/SOF) and ‘ΔHCV core antigen’ were closely associated with the ΔLDL-C values. Of these two factors, IFN-free regimen (LDV/SOF) was most closely associated with the ΔLDL-C level (standardized β, 0.39; p<0.01).

**Table 3:** Factors Associated with ΔLDL-C value with HCV administered IFN-free regimen, Analyzed by Linear Regression Analysis.

Characteristics	Univariate analysis			Multivariate analysis		
	standardized β	adjusted R^2^	P-value	standardized β	adjusted R^2^	P-value
IFN-Free Regimen (DCV/ASV:0, LDV/SOF:1)	0.44	0.18	<0.01	0.39	0.21	<0.01
ΔHCV core antigen (D0-D1) (log_10_(fmol/L))	0.31	0.08	<0.01	0.21		0.04
TG (mg/dl)	0.25	0.05	0.02	−	−	−
Fib-4 index	-0.22	0.04	0.05	−	−	−
Age, year	−	−	0.84	−	−	−
Sex (female:0, male:1)	−	−	0.25	−	−	−
BMI (kg/m^2^)	−	−	0.42	−	−	−
HCV-RNA (D0) (log_10_IU/ml)	−	−	0.45	−	−	−
HCV core antigen (D0) (log_10_(fmol/L))	−	−	0.45	−	−	−
AST (IU/L)	−	−	0.31	−	−	−
ALT (IU/L)	−	−	0.93	−	−	−
γ-GTP (IU/L)	−	−	0.29	−	−	−
Alb (g/dl)	−	−	0.35	−	−	−
Platelet count (×10^3^/μl)	−	−	0.06	−	−	−
TC (mg/dl)	−	−	0.69	−	−	−
LDL-C (mg/dl)	−	−	0.65	−	−	−
AFP (ng/ml)	−	−	0.18	−	−	−
ΔHCV-RNA (D0-D1) (log_10_IU/ml)	−	−	0.34	−	−	−

### The Relationship between ΔLDL-C and ΔHCV Core Antigen

**Fig 2:**
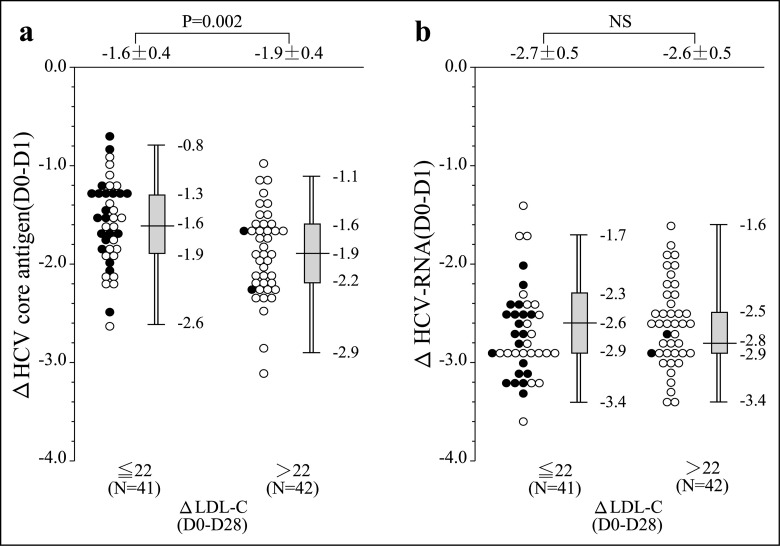
Comparison of serum HCV dynamics of patients after starting IFN-free treatment stratified by ΔLDL-C level. (a) The decrease of HCV core antigen was more rapid in the patients with higher ΔLDL-C values than in those with low ΔLDL-C. (b) There was no difference in the decline of HCV-RNA regardless of the value of ΔLDL-C. Open circles: the LDV/SOF patients. Closed circles: the DCV/ASV patients.

**Fig 3:**
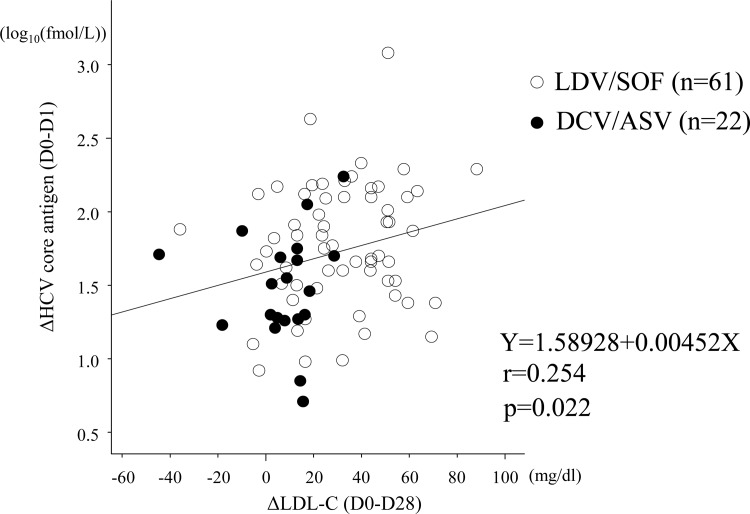
Correlation between ΔLDL-C (D0-D28) and ΔHCV core antigen (D0-D1). Scatterplots with fitting line show positive correlation between ΔLDL-C and ΔHCV core antigen. Pearson’s correlation provides coefficient (r) and p-value. Open circles: the LDV/SOF patients. Closed circles: the DCV/ASV patients.

Fig 2 shows the relationship between the ΔLCL-C and the HCV viral decline. There was no difference in the decline of HCV-RNA regardless of the value of ΔLDL-C (Fig 2B). On the other hand, the decrease of HCV core antigen was more rapid in the patients with higher ΔLDL-C values than in those with low ΔLDL-C (Fig 2A). The more the HCV core antigen values decreased, the more the LDL-C values rose, especially in the LDV/SOF-treated patients and in the high-ΔLDL-C patients. Fig 3 shows the correlation between ΔLDL-C (D0-D28) and ΔHCV core antigen (D0-D1). Scatterplots with fitting line show positive correlation between ΔLDL-C and ΔHCV core antigen (r = 0.254, p = 0.022).

## Discussion

In this study, we observed a rapid increase in serum LDL-C and TC in HCV-infected Japanese patients between baseline and treatment day 28 under the treatment of their HCV infection with two types of IFN-free DAA therapy: DCV/ASV combination therapy and LDV/SOF combination therapy. Our results showed that the increases in serum LDL-C and TC during HCV therapy were strongly dependent on the type of HCV therapy, and that there was an apparent difference between these two therapies. This is the first report to demonstrate that DCV/ASV combination therapy resulted in weaker increases in serum LDL-C and TC compared to LDV/SOF combination therapy.

Although we cannot yet make a conclusion as to why there was an apparent difference in the kinetics of serum LDL-C and TC provided by the DCV/ASV and LDV/SOF therapies, we are considering the following two reasons. (1) There may be a difference in the mechanism of anti-viral action between the two therapies. DCV and LDV are both NS5A inhibitors [16,18], and the difference in the therapies is that ASV is a protease inhibitor and SOF is a polymerase inhibitor [16,18]. (2) SOF itself may act directly to rapidly raise the serum LDL-C level.

Although DCV/ASV combination therapy is used widely in Japan, we have been unable to find studies that reported rapid increases in LDL-C and TC during at the early period of therapy. However, we found two reports that serum LDL-C and TC increased during SOF treatment. In 2015, Meissener et al. were the first to report a rapid and sustained increase in the LDL-C concentration in HCV, GT1-monoinfected patients treated for 24 weeks with SOF/RBV [20]. The LDL-C changes were sustained post-treatment in the patients who achieved an SVR, whereas the LDL-C declined to pretreatment levels in the patients who experienced treatment relapse [20]. Those authors noted that a marked increase in serum LDL-C was correlated with a viral decline in serum, suggesting a direct influence of HCV clearance on serum cholesterol [20].

Townsend et al. later reported a rapid increase in both LDL-C and TC values that was sustained during and after treatment in HIV/HCV-co-infected patients treated with LDV/SOF combination therapy for 12 weeks [21]. They found that there was no difference in the increase in serum LDL-C and TC between their HIV/HCV-co-infected and HCV mono-infected patients [21]. The Meissener and Townsend studies both confirmed rapid increases in LDL-C and LDL particle size by week 2 or week 4 of treatment that was sustained during and after treatment by performing an NMR (nuclear magnetic resonance) LipoProfile analysis using patient serum to quantify the absolute number of distinct lipoparticles in serum by size [20,21].

In the present study, we examined two types of HCV-RNA replication markers (HCV-RNA and HCV core antigen) to investigate the connection between intrahepatic HCV suppression and the increase in LDL-C during IFN-free treatment at the early period of therapy. Our comparison of the disappearance rate of serum HCV-RNA of the start of therapy to that at therapy day 28 between DCV/ASV combination therapy and LDV/SOF combination therapy revealed no significant difference of the disappearance rate of serum HCV-RNA between the two groups (LDV/SOF 87.5% vs. DCV/ASV 80.5%). There was also no significant difference in the decline of the serum level of HCV-RNA over days 0–1 between the LDV/SOF-treated patients (−2.6 log IU/ml/day) and the DCV/ASV-treated patients (−2.7 log IU/ml/day). However, interestingly, our analysis revealed that the decline level of HCV core antigen during days 0–1 was associated with a rapid increase in serum LDL-C. Another interesting result was that the day 0–1 drop in the HCV core antigen level in the LDV/SOF group (−1.8 log fmol/L/day) was significantly greater than that in the DCV/ASV group (DCV/ASV: −1.5 log fmol/L/day; p = 0.02).

The lipid biogenesis of the host is closely related to the RNA replication and assembly of hepatitis C virus particles. HCV core protein localizes to the surface of lipid droplets and this localization plays a critical role in HCV particle assembly and it contributes to the accumulation and production of host lipid components [23,24]. Herker E etal. identified the triglyceride-synthesizing enzyme DGAT1 as an important host factor for HCV infection; DGAT1 interacts with the viral nucleocapsid core and is required for the trafficking of core to lipid droplets [25]. Our present findings supported the concept that the suppression of HCV core protein production by IFN- free treatment reflects the reduction in the production of lipid droplets in HCV-infected liver cells, resulting in a rebound of circulating LDL-C and TC.

The important role of lipid droplets might be associated with the production of “infectious” HCV [24]. It's confirmed that two kinds of HCV particle in HCV-infected cultured cells. They are minor low-density particles (approx. 1.12 g/ml) with high infectivity and major high-density particles (approx. 1.15 g/ml) without infectivity. The difference in density and infectivity between two kinds of HCV particles, may be due to the lipid composition. Cholesterol and sphingolipids are important for virion maturation and infectivity, as cholesterol-depleted or sphingomyelin-hydrolysed virus negatively impact infectivity [24]. In the present study, the significantly larger reduction of the day 0–1 drop in the HCV core level during the LDV/SOF therapy compared to the DCV/ASV therapy might indicate that LDV/SOF combination therapy would be effective for the eradication of infectious HCV, although the size of the decrease in HCV RNA was not significantly different between these two therapies.

Our findings—obtained from a substantial number of patients with meaningful increases in serum LDL-C and TC induced by IFN-free treatment—have important implications for hyperlipidemia management [26]. Imano et al. assessed the association between serum LDL-cholesterol levels and the risk of coronary heart disease (CHD) among Japanese in the Circulatory Risk in Communities Study (CIRCS) [27]. The CIRCS results showed that the subjects with an LDL-C level ≥140 mg/dL had the risk values of 2.80 for total CHD, 3.83 for myocardial infarction, 4.07 for non-fatal CHD, and 1.24 for fatal CHD compared to the subjects with an LDL-C level <80 mg/dL [27]. Previous studies demonstrated the benefit of lipid lowering for the primary prevention of CHD [27–31].

As HCV continues to be eradicated with increasing frequency among HCV-infected subjects, the post-treatment viral clearance is associated with increased LDL-C and cholesterol, often to levels associated with an increased risk of CHD [21,26,32]. We suggest that among patients undergoing successful antiviral therapy, serum lipid levels should be assessed in a careful follow-up, as clearance may reveal some patients with previously unappreciated coronary risk. Additional prospective studies of large patient cohorts are needed to further examine the correlation between the rise in lipid levels and clinically significant outcomes, such as the development of CHD.

Our study has at least two limitations. The limited number of cases (n = 100) is the first. The other is that we did not conduct an analysis that covered a longer term, and it is of interest to know whether high LDL-C levels continue after the completion of the treatments used in this population.

In conclusion, our study revealed that a rapid increase in the serum LDL concentration during the IFN-free treatment of hepatitis C patients was associated with the type of HCV therapy and a decline in the level of HCV core protein. In HCV-infected patients undergoing IFN-free treatment for HCV, lipids should be monitored post-treatment for a cardiovascular risk assessment and to determine the presence of indications for lipid-modulating therapy.

## Supporting Information

S1 Table Analysis data set. All patients data sets were included in the following file; “LDL-C analysis, DCVASV and LDVSOF n = 100. xlsx”.
(XLSX)
